# First report of accumulation of perfluorooctane sulfonate (PFOS) in platypuses *(Ornithorhynchus anatinus)* in New South Wales, Australia

**DOI:** 10.1007/s11356-024-34704-w

**Published:** 2024-08-16

**Authors:** Katherine G. Warwick, Ian A. Wright, Jessica Whinfield, Jason K. Reynolds, Michelle M. Ryan

**Affiliations:** 1 School of Science, Western Sydney University, Locked Bag 1797, Penrith, NSW 2751 Australia; 2 Taronga Conservation Society, Sydney, Australia; 3 Harry Butler Institute, Murdoch University, Murdoch, Australia

**Keywords:** Aquatic ecology, Bioaccumulation, Contamination, PFOS, Pollution, Platypus

## Abstract

The platypus (*Ornithorhynchus anatinus*) is a semi-aquatic monotreme that occupies a high trophic position in the freshwater ecosystems of eastern mainland Australia and Tasmania. Platypuses are continuously exposed to anthropogenic contaminants including perfluorooctane sulfonate (PFOS). This study examined PFOS concentrations in the livers of deceased platypuses (eight wild; one captive) that were opportunistically collected across NSW over a two- and a half-year period. There was a large variation in PFOS concentrations, ranging from < 1 µg/kg to 1200 µg/kg. This study presents the first report of PFOS contamination in platypuses, revealing their PFOS levels are broadly similar to those found in river otters (*Lutra canadensis*) and lower than those in American mink (*Mustela* vison), both which occupy similar ecological niches in freshwater systems. This study raises concerns about the impact of PFOS on platypus health.

## Introduction

The platypus (*Ornithorhynchus anatinus*) is endemic to many rivers and streams in eastern Australia. However, there are growing concerns for their conservation with reports of declining abundance and distribution, including local extirpations (Woinarski et al. 2014). The species is recognised as having a declining population and is listed as “Near Threatened’ by the International Union for Conservation of Nature (IUCN) (Woinarski and Burbidge 2016). Platypuses are vulnerable to many impacts associated with human activity including hydrological changes, decline in water quality, increase in litter and discarded fishing line, illegal opera house nets (yabby traps) and water contamination (Grant and Temple-Smith 2003; Serena et al. 2016). Anthropogenic contaminants have the potential to enter waterways and disrupt aquatic ecosystems. Serena and Pettigrove (2005) found a negative correlation between heavy metal contaminants in sediment and platypus population abundance and a previous study by Munday et al. (2002) found persistent organic pollutants in platypus. One anthropogenic persistent waterway contaminant is perfluorooctane sulfonate (PFOS) (Butt et al. 2010) a homologue of PFAS and is defined by having an eight-carbon fluorocarbon chain with a sulfonate acid functional group. PFOS does not readily biodegrade and can persist in the environment for years (Nicole 2020) and has been reported to enter aquatic food chain and potentially bioaccumulate (Kannan et al. 2002; O’Rourke et al. 2022, 2024; Well et al. 2024). To date there have been no studies that examined PFOS concentrations in platypus tissue.

Previous studies of European otters (*lutra lutra) (*O’Rourke et al. 2022, 2024), Northern American river otters (*Lutra canadensis)* and mink (*Mustela vison*) (Kannan et al. 2002) all of which occupy a similar ecological niche, have examined PFOS concentration in liver samples, as such this study also chose to analyse liver samples for comparison. Health concerns for aquatic wildlife from exposure to high concentrations of PFOS include increased liver weight, decreased thyroid function, decreased immunity and neurological disorders (Keller et al. 2012). There are no current detected concentrations that are considered safe for platypus health, however draft guidelines by the Australian government suggest that exposure directly from their diet should not exceed 3.1 µg/kg of wet weight (combined PFOS and PFHxS concentrations) (DCCEEW 2022).

Theoretically, platypuses may be exposed to high levels of PFOS. PFOS can bioaccumulate, and the food source of platypuses, aquatic invertebrates, have been reported to contain substantial PFOS levels (Ahrens and Bundschuh 2014). Platypuses consume up to 21% of their body mass daily, and up to 36% of body mass in lactating females, of aquatic invertebrates (Thomas et al. 2020). Additionally, it is known that aquatic environments have the greatest risk of PFOS contamination, due to surface run off and effluent discharge (Australia and New Zealand guidelines for fresh and marine water quality n.d.). Many studies have documented the accumulation of PFOS in vertebrate species, however these studies focused primarily on livestock, or marine mammals and birds (Ahrens and Bundschuh 2014; Foord et al. 2024). For example, a recent study in Australia examined little penguin (*Eudyptula minor)* scats, eggs and plasma and found 14 homologue of PFAS with PFOS being the most commonly detected (Well et al. 2024). This study found a positive correlation between PFOS concentration and urbanised environments (Well et al. 2024).

American mink and river otters are two of the only freshwater mammal species that have been assessed for PFOS levels, and both are regarded as sentinel species for detecting environmental contaminants in aquatic systems (Kannan et al. 2002). They also occupy a similar ecological niche to the platypus: all three live in freshwater environments and are predators at the top of the aquatic ecosystem. Toxicology studies in mink and otters have recorded very high liver PFOS concentrations (20–5140 µg/kg and 25–994 µg/kg, respectively) (Kannan et al. 2002), with investigations in mink reporting some of the highest PFOS concentrations detected in freshwater sentinel species (Ahrens and Bundschuh 2014). The biomagnification of PFOS by mink was confirmed by a controlled captive feeding study (Ahrens and Bundschuh 2014). The aim of this study was to determine if PFOS were present in the livers of platypuses and if so, at what concentration. If platypuses are consuming PFOS directly from their food source, then it would be expected that platypuses with a lower TVI (higher body fat percentage) would have a higher concentration of PFOS owing to a larger dietary intake compared with platypuses with a higher TVI (lower body fat percentage) (Macgregor et al. 2016). This was achieved by opportunistically collecting and testing samples from incidentally deceased platypuses from New South Wales (NSW).

## Materials and methods

**Fig.1:**
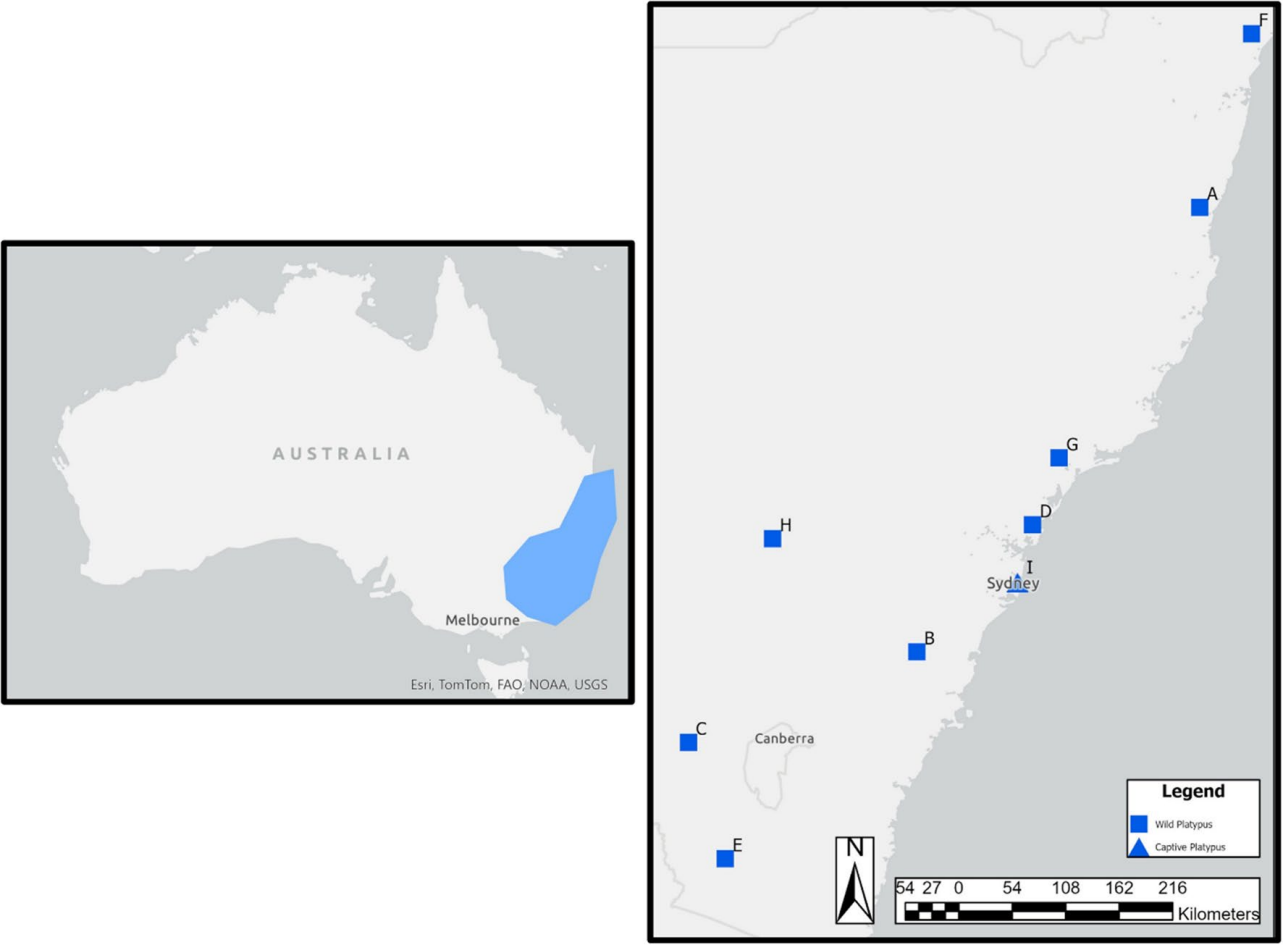
Location of platypus carcasses collected across New South Wales, Australia

Platypus carcasses (n = 9) were collected between 2020 and 2023 from nine locations across NSW (Fig. 1). Of these, eight were from the wild and one from captivity. Liver samples were collected at the same time as necropsy was performed, which for four platypus carcasses (A, D, F and I) were performed within 24 h of death. Five of the platypuses (B, C, E, G and H) were stored in a -20 °C freezer for up to 10 months until a necropsy was performed. For each carcass, a gross necropsy and sample collection was performed at Taronga Zoo, Sydney, by a wildlife veterinarian. The following variables were recorded: Tail volume index (TVI), degree of decomposition, length (tip of bill to end of tail), weight, sex, and age (based on spur morphology) (Grant and Carrick 1978; Williams et al. 2012) (Table 1). A platypus stores approximately 50% of its total fat volume in its tail and as such is TVI is the current industry standard for assessing body condition (Macgregor et al. 2016). A TVI of 1 indicates high fat deposits in the tail and a TVI of 5 indicates emaciation. TVI is assessed by squeezing the edges of the tail together, the closer the edges are to touching each other, the lower the fat deposits in the tail.

**Table 1:** Summary of platypus data. TVI is defined in the methods and materials. Age refers to juveniles (< 12 months), sub-adults (13–24 months) adults (> 24 months)

Platypus	PFOS liver concentration (µg/kg)	Tail Volume Index (TVI)	Weight (grams)	Length (mm)	Sex, age	Wild/ Captive	Cause of death	State of decomposition	Location (river, nearest township)
A	4	4	1105	480	Male, adult	Wild	Neurological disease, unknown aetiology	Fresh	Bellinger River, Bellingen
B	390	2	1420	520	Male, adult	Wild	Drowning due to fishing gear	Mild/moderate	Wingecarribee River, Berrima
C	10	3	1653	510	Male, adult	Wild	Drowning due to fishing gear	Mild/moderate	Tumut River, Tumut
D	740	1	671	370	Female, sub-adult	Wild	Open	Advanced	Ourimbah Creek, Ourimbah
E	5	2	682	400	Male, juvenile	Wild	Drowning due to fishing gear	Mild/moderate	Thredbo River, Jindabyne
F	19	3	430	318	Male, juvenile	Wild	Vehicle trauma	Fresh	Marom Creek, Marom Creek
G	1200	1	1170	460	Male, juvenile	Wild	Drowning due to fishing gear	Advanced	Hunter River, Morpeth
H	17	3	1578	540	Male, adult	Wild	Vehicle trauma	Mild/moderate	Unnamed Creek, Orange
I	< 1	3	728	395	Female, adult (26 years)	Captive	Pulmonary oedema, unknown aetiology	Fresh	Taronga Zoo, Sydney

Liver samples were used to test for PFOS due to previously published studies of European otters (*Lutra lutra*) and Northern American mink (*Mustela vison*) and river otters (*Lutra* *canadensis*) that also used liver (Kannan et al. 2002). Given that these species occupy a similar ecological niche, these results are comparable. The livers were wrapped in aluminium foil and stored at -20 °C prior to analysis. The samples were prepared for analysis at the Western Sydney University Hawkesbury Laboratory. Livers were freeze dried at -40 °C over two 18-h cycles using an Edwards Freeze Dryer Modulyo with a Pirani 501 vacuum gauge control. Freeze dried samples were then analysed at EnviroLab, Chatswood, Sydney, (National Association of Testing Authorities accredited) for concentrations of PFOS using solid phase extraction and liquid chromatography tandem mass spectrometry. Due to the freeze-drying process and minimum sample weight required for laboratory analysis (1 g), only a single replicate of liver could be obtained from each platypus. Duplicate samples and matrix spike recoveries were analysed at a frequency to meet or exceed NEPM requirements. The duplicate sample, relative percentage difference (RPD) and matrix spike recoveries for the batch were within the laboratory acceptance criteria.

## Results and discussion

This study detected concentration of PFOS in eight of the nine individual platypus livers, ranging from 4–1200 µg/kg. The only liver that did not result in detectable PFOS concentrations came from the only captive platypus in the study (Table 1). There are no guidelines on what constitutes safe concentrations of PFOS in wildlife.

In the case with the highest recorded concentration of PFOS (1200 µg/kg, platypus G) the location of origin had no reporting of water testing for PFOS nor is there any publicly available documentation of PFOS contamination based on the NSW Government PFAS Response website (DPI&RD 2024) and NSW EPA (NSW Epa 2024). However, in the upstream catchment (up to 110 km^2^) of where the platypus was found, there is a wastewater treatment plant, and a regional airport. Additionally, a fire station was in the immediate vicinity of both this case and the case with the third highest concentration of PFOS (390 µg/kg, platypus B). Whilst this study did not investigate the source of PFOS contamination, research has shown increased concentrations of PFOS associated with airports, and firefighting locations including training facilities (Australian and New Zealand Guidelines fresh and marine quality n.d.; United Nations Environment Programme (2006). The only platypus in this study that had undetectable levels of PFOS was the captive platypus (I). This suggests that the provision of filtered water may reduce the likelihood of PFOS accumulation.

**Fig.2:**
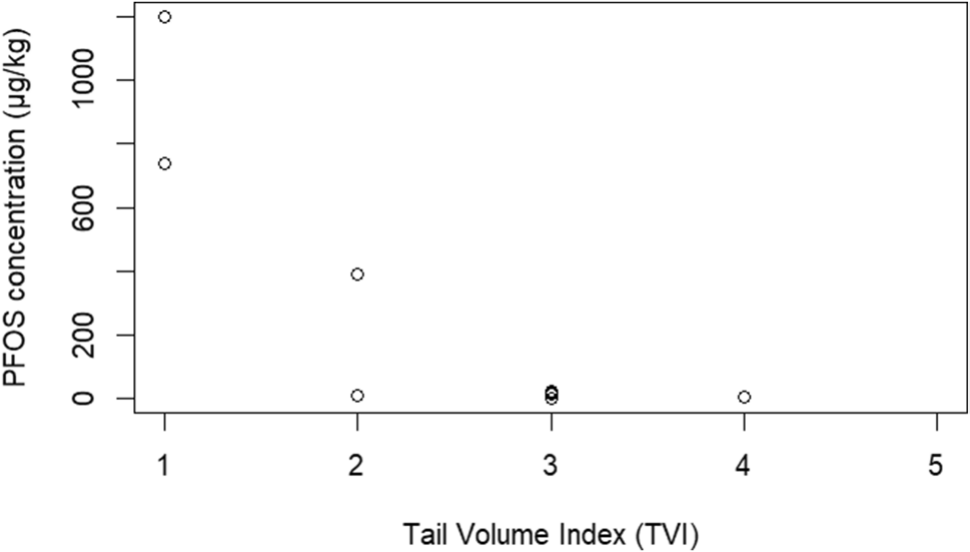
Relationship between Tail Volume Index (TVI) and PFOS concentration (µg/kg) in the liver

The results of this study show a negative relationship between liver PFOS concentration and body condition, as assessed by the Tail Volume Index (TVI) (Fig. 2). This study found that platypuses with the lowest TVI, and thus best body condition (D and G), had the highest concentrations of PFOS (Table 1). This observed negative relationship could be a result of platypuses in better body condition consuming a higher daily biomass, and therefore being exposed to a higher concentration of PFOS through their food source. This study found no relationship between PFOS concentration and age, sex, total body weight, and/or total body length.

A limitation of this study is that it was not possible to replicate or control external factors. Deceased platypuses were collected opportunistically from across NSW and therefore factors including location, water quality, age, sex, and degree of decomposition could not be controlled. Due to the nature of this sampling method, it was also not possible to have a control or reference sample, although given the widespread nature of PFOS it is unlikely that there is a wild platypus population that is completely unaffected by the synthetic chemical.

The results of this study show that platypuses are accumulating PFOS in very high concentrations, at comparable levels to those previously recorded in river otters, but less than what was previously recorded in mink (Kannan et al. 2002). Given the small sample size of this study, only observed concentrations of PFOS were reported on, further research should include statistical analysis to determine if a correlation between TVI and PFOS concentration could be determined and if so, what this means. Future studies should explore health impacts associated with exposure to PFOS and the direct and indirect bioaccumulation pathways.

## Ethical approval

This project operated under Western Sydney University Biosecurity and Radiation approval (B14275), New South Wales National Parks and Wildlife Service Scientific permit (SL102542), and Taronga Conservation Society opportunistic sampling request agreement (R22D343).

## Data Availability

Data sets generated during the current study are available from the corresponding author upon reasonable request.

## References

[CR1] Ahrens L, Bundschuh M (2014) Fate and effects of poly- and perfluoroalkyl substances in the aquatic environment: A review. Environ Toxicol Chem 33:1921–1929 24924660 10.1002/etc.2663

[CR2] Australian and New Zealand Guidelines for Fresh and Marine Water Quality (2000) Toxicant default guideline values for aquatic ecosystem protection: Perfluorooctane sulfonate (PFOS) in freshwater. Australian and New Zealand Governments and Australian state and territory governments, Canberra, ACT, Australia: Available at https://waterquality.gov.au/anz-guidelines/guideline-values/default/water-qualitytoxicants/toxicants

[CR3] DPI&RD (2024) PFAS response. NSW Department of Primary Industry and Regional Development. Available at: https://www.dpi.nsw.gov.au/biosecurity/pfas-response

[CR4] Butt CM, Berger U, Bossi R, Tomy GT (2010) Levels and trends of poly- and perfluorinated compounds in the arctic environment. Sci Total Environ 408(15):2936–2965 20493516 10.1016/j.scitotenv.2010.03.015

[CR5] Foord CS, Szabo D, Robb K, Clarke BO, Nugegoda D (2024) Hepatic concentrations of per- and polyfluoroalkyl substances (PFAS) in dolphins from south-east Australia: Highest reported globally. Sci Total Environ 908:168438 37963535 10.1016/j.scitotenv.2023.168438

[CR6] Grant TR, Carrick NF (1978) Some aspects of the ecology of the platypus, *Ornithorhynchus anatinus*, in the upper Shoalhaven River, New South Wales. Aust J Zool 20:181–199

[CR7] Grant TR, Temple-Smith P (2003) Conservation of the Platypus, Ornithorhynchus anatinus - Threats and Challenges. Aquat Ecosyst Health Manag 6(1):5–18 10.1080/14634980301481

[CR8] Kannan K, Newsted J, Halbrook RS, Giesy JP (2002) Perfluorooctanesulfonate and related fluorinated hydrocarbons in mink and river otters from the United States. Environ Sci Technol 36:2566–2571 12099451 10.1021/es0205028

[CR9] Keller JM, Ngai L, McNeill JB, Wood LD, Stewart KR, O’Connell SD, Kucklick JR (2012) Perfluoroalkyl contaminants in plasma of five sea turtle species: Comparisons in concentration and potential health risks. Environ Toxicol Chem 31(6):1223–1230 22447337 10.1002/etc.1818

[CR10] Macgregor JW, Holyoake C, Munks S, Connolly JH, Robertson ID, Fleming PA, Lonsdale RA, Warren K (2016) Assessing body condition in the Platypus (*Ornithorhynchus anatinus):* a comparison of old and new methods. Aust J Zool 64(6):421–429 10.1071/ZO16071

[CR11] Munday BL, Stewart NJ, Södergren AS (2002) Accumulation of persistent organic pollutants in Tasmanian platypus (*Ornithorhynchus anatinus*). Environ Pollut 120(2):233–237 12395834 10.1016/S0269-7491(02)00143-4

[CR12] Nicole W (2020) Breaking It Down: Estimating Short-Chain PFAS Half-Lives in a Human Population. Environ Health Perspect 128(11):114002 33174763 10.1289/EHP7853 PMC7657368

[CR13] NSW EPA (2024) The NSW Government PFAS Investigation Program. NSW Environment Protection Authority. Available at: https://www.epa.nsw.gov.au/your-environment/contaminated-land/pfas-investigation-program

[CR14] O’Rourke E, Hynes J, Losada S, Barber JL, Gloria Pereira M, Kean EF, Hailer F, Chadwick EA (2022) Anthropogenic Drivers of Variation in Concentrations of Perfluoroalkyl Substances in Otters (*Lutra lutra*) from England and Wales. Environ Sci Technol 56(3):1675–1687 35014794 10.1021/acs.est.1c05410 PMC8812117

[CR15] O’Rourke E, Losada S, Barber JL, Scholey G, Bain I, Gloria Pereira M, Hailer F, Chadwick EA (2024) Persistence of PFOA Pollution at a PTFE Production Site and Occurrence of Replacement PFASs in English Freshwater Revealed by Sentinel Species, the Eurasian Otter (*Lutra lutra*). Environ Sci Technol 58(23):10195–10206 38800846 10.1021/acs.est.3c09405 PMC11171452

[CR16] Serena M, Pettigrove V (2005) Relationship of sediment toxicants and water quality to the distribution of platypus populations in urban streams. North Am Benthol Soc 24:679–689 10.1899/04-024.1

[CR17] Serena M, Grant TR, Williams GA (2016) Reducing bycatch mortality in crustacean traps: effect of trap design on platypus and yabby retention rates. Fish Res 175:43–50 10.1016/j.fishres.2015.11.010

[CR18] Thomas JL, Parrott ML, Handasyde KA, Temple-Smith P. (2020) Maternal care of platypus nestlings (*Ornithorhynchus anatinus*). Aust Mammal 42:283–292

[CR19] United Nations Environment Programme (2006) Stockholm Convention on Persistent Organic Pollutants Persistent Organic Pollutants Review. Committee Second meeting Geneva, 6–10 November 2006 Report of the Persistent Organic Pollutants Review Committee on the work of its second meeting. Addendum. Risk profile on perfluorooctane sulfonate.

[CR20] Well MR, Coggan TL, Stevenson G, Singh N, Askeland M, Lea M, Philips A, Carver S (2024) Per- and polyfluoroalkyl substances (PFAS) in little penguins and associations with urbanisation and health parameters. Sci Total Environ 912:169084. 10.1016/j.scitotenv.2023.169084 38056658 10.1016/j.scitotenv.2023.169084

[CR21] Williams GA, Serena M, Grant TR (2012) Age-related change in spurs and spur sheaths of the platypus (*Ornithorhynchus anatinus)*. Aust Mammal 35(1):107–114 10.1071/AM12011

[CR22] Woinarski JCZ, Burbidge AA, Harrison PL (2014) The Action Plan for Australian mammals 2012. CSIRO Publishing, Collingwood

[CR23] *Woinarski JCZ, Burbidge AA (2016).* Ornithorhynchus anatinus. The IUCN Red List of Threatened Species 2016:e.T40488A21964009. 10.2305/IUCN.UK.2016-1.RLTS.T40488A21964009.en

